# Endocrine Dysfunctions After Pediatric Traumatic Brain Injury: Present Insights and Future Directions

**DOI:** 10.3390/children12111484

**Published:** 2025-11-03

**Authors:** Ignazio Cammisa, Elena Malavolta, Giorgio Sodero, Donato Rigante, Clelia Cipolla

**Affiliations:** 1 Department of Life Sciences and Public Health, Fondazione Policlinico Universitario A. Gemelli IRCCS, 00168 Rome, Italy; elena.malavolta01@icatt.it (E.M.); donato.rigante@unicatt.it (D.R.); clelia.cipolla@policlinicogemelli.it (C.C.); 2 Pediatric Department, Perrino Hospital, 72100 Brindisi, Italy; giorgio.sodero01@icatt.it; 3 Pediatric Endocrinology Unit, Perrino Hospital, 72100 Brindisi, Italy; 4 Università Cattolica Sacro Cuore, 00168 Rome, Italy

**Keywords:** traumatic brain injury, endocrine dysfunction, children, hypothalamic–pituitary axis, growth hormone deficiency, puberty

## Abstract

Traumatic brain injury (TBI) in childhood is a major global health concern and a leading cause of morbidity and mortality in the pediatric population. Its incidence is rising worldwide, with early childhood and adolescence representing the most vulnerable age groups. Beyond acute neurological injury, post-traumatic endocrine dysfunction has emerged as an underrecognized but clinically significant sequela, with potential long-term consequences for growth, puberty, metabolism, and overall quality of life. The hypothalamic–pituitary axis (HPA) is uniquely vulnerable due to its anatomical and vascular characteristics, making pituitary cells—particularly somatotrophs and gonadotrophs—susceptible to ischemic, traumatic, and inflammatory damage. Reported prevalence of post-TBI pituitary dysfunction in children ranges from 5 to 57%, reflecting a deep heterogeneity in injury severity, diagnostic methods, and timing of evaluations. Growth hormone deficiency (GHD) is the most frequently reported abnormality, with presentations varying from transient to persistent forms. Gonadal axis disturbances, including hypogonadotropic hypogonadism and, less commonly, central precocious puberty, highlight the impact of TBI on pubertal development. Adrenal dysfunctions, though less frequent, may be life-threatening if unrecognized, while posterior pituitary disorders, such as diabetes insipidus, usually revealed acutely, are often transient. Importantly, many endocrine sequelae manifest months to years after the initial trauma, complicating a timely diagnosis. Current evidence underscores the need for structured, longitudinal endocrine surveillance after pediatric TBI, with baseline and follow-up assessments at defined intervals. Early recognition and intervention, including hormone replacement when appropriate, may improve neurocognitive recovery and overall rehabilitation outcomes. Future multicenter studies and standardized screening protocols should be considered essential to clarify incidence, natural history, and optimal management strategies for post-traumatic endocrine dysfunction in children.

## 1. Introduction

**Figure 1:**
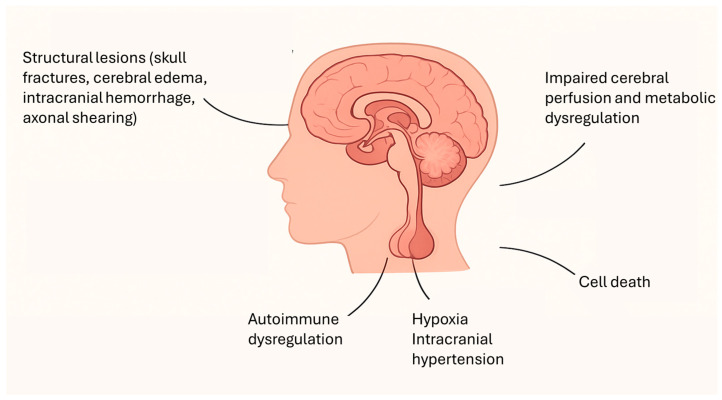
Key mechanisms contributing to hypothalamic–pituitary axis dysfunction after traumatic brain injury.

Traumatic brain injury (TBI) in childhood represents a significant global health issue and continues to rank among the leading causes of morbidity and mortality within the pediatric population [1,2,3]. Its incidence has been steadily rising, with annual estimates ranging between 180 and 250 cases per 100,000 children. Two age groups appear particularly vulnerable: early childhood, where falls represent the predominant etiology, and adolescence, when sports-related trauma, bicycle accidents, road traffic incidents, and risk-taking behaviors constitute the main contributors [2,3,4]. Each year, approximately 29,000 children aged 0–14 years require hospitalization for TBI, of whom nearly 10% sustain moderate to severe injuries. Despite this considerable burden, the acute and chronic pathophysiological processes underlying pediatric TBI—and the spectrum of long-term consequences—remain incompletely understood [1,2,3,4,5,6]. Among these sequelae, post-traumatic endocrine dysfunction has emerged as a clinically relevant yet frequently underrecognized complication. Beyond the immediate structural and functional cerebral damage, TBI can trigger substantial neuroendocrine alterations, often manifesting days to months after the initial insult [5,6,7]. The pathophysiology of TBI is complex and multifactorial, encompassing not only the primary mechanical injury but also a cascade of secondary processes, including systemic hypotension, hypoxia, intracranial hypertension, and disturbances in cerebral perfusion and metabolism, as reported in Figure 1 [7,8]. Structural lesions such as skull fractures, cerebral edema, and intracranial hemorrhage further exacerbate intracranial pressure and can contribute to hypothalamic–pituitary axis (HPA) injuries, in addition to direct axonal shearing [8]. The primary injury is characterized by impaired cerebral perfusion, metabolic dysregulation, and ionic imbalance, ultimately leading to edema and cell death. Secondary injury, evolving over hours to weeks, aggravates this damage through vascular compromise and widespread central nervous system (CNS) swelling, with potentially fatal outcomes such as brainstem herniation [5]. In addition to these mechanical and vascular mechanisms, growing evidence suggests an autoimmune component in the pathogenesis of post-traumatic hypopituitarism. Tanriverdi et al. demonstrated that anti-pituitary antibodies can persist for up to three years in patients with TBI-induced pituitary dysfunction, thereby supporting the hypothesis that immune-mediated mechanisms may significantly contribute to chronic pituitary impairment [9,10].

Regardless of the underlying mechanism, the HPA occupies a uniquely vulnerable anatomical and physiological position. Enclosed within the rigid sella turcica, the pituitary gland is tethered by the delicate stalk and sustained by a fragile portal microvascular system, both highly sensitive to fluctuations in cerebral perfusion [11,12,13]. Dysfunction of the anterior pituitary is observed more frequently than involvement of the posterior lobe following pediatric traumatic brain injury, a discrepancy largely explained by differences in vascular anatomy and vulnerability to injury. The posterior pituitary (neurohypophysis) is perfused directly by the short hypophyseal arteries, providing relatively robust blood flow and conferring resilience to ischemic or traumatic insults. In contrast, the anterior pituitary (adenohypophysis) relies predominantly on the long hypophyseal vessels that form the hypothalamo–hypophyseal portal system, which courses through the pituitary stalk and branches into an extensive network of low-pressure portal capillaries. This indirect, low-pressure vascular supply renders the anterior lobe especially susceptible to ischemia, shearing forces, and hypoxic injury, particularly in the setting of traumatic axonal injury, edema, or elevated intracranial pressure [11,12,13]. Within the anterior pituitary, somatotroph and gonadotroph cells—located peripherally—appear especially susceptible to injury, whereas corticotroph and thyrotroph cells, located more centrally, are relatively preserved [11,12].

The reported incidence of HPA dysfunction following pediatric TBI is highly heterogeneous, reflecting differences in injury severity, timing of endocrine assessment, and diagnostic methodologies. The absence of standardized screening protocols and consensus guidelines likely contributes to underrecognition, with a median diagnostic delay of five years after injury, thereby underestimating the true prevalence [14,15]. In pediatric cohorts, the prevalence of post-traumatic pituitary dysfunction has been reported to range from 5% to 57%, with estimates increasing to 86% in studies that considered ‘hyperprolactinemia’ as an abnormality [16]. A systematic review by Lauzier et al., encompassing 66 studies and 5386 adult patients, analyzed anterior pituitary dysfunction across different time intervals: less than 3 months, 3–12 months, and more than 12 months post-injury. They reported hypopituitarism in 45% of patients in the acute phase, 36% in the subacute phase, and 32% in the chronic phase. Risk factors associated with higher prevalence included older age, more severe injury, and skull fractures [17]. Data from pediatric studies remain equally variable. In a mixed retrospective and prospective study of 48 children with TBI, Einaudi et al. reported HPA dysfunction in 10% of cases at 12 months, while Niederland et al., in a cross-sectional analysis of 26 children assessed at 30 ± 8 months, observed pituitary abnormalities in 61% [18,19]. Similarly, Poomthavorn et al. identified pituitary dysfunction in 17% of patients, whereas Norwood et al. reported isolated growth hormone deficiency (GHD) in 16% [2,20]. In contrast, two cross-sectional investigations published in 2010 failed to detect clinically relevant endocrine abnormalities in children following TBI, suggesting that under certain clinical conditions or within specific cohorts, measurable hormonal dysfunction may not invariably occur [21,22]. Although the underlying pathophysiological mechanisms are increasingly well characterized, the clinical presentation of endocrine sequelae is often delayed and insidious, in contrast to overt and acute manifestations of neurological injuries. This diagnostic challenge frequently results in underrecognition and delayed treatment, with potential long-term implications for growth, puberty, metabolism, and overall quality of life.

This review aims to provide a comprehensive synthesis of current evidence regarding endocrine disturbances following pediatric TBI, with a particular focus on HPA dysfunction.

## 2. Materials and Methods

We conducted a comprehensive literature review to explore the endocrine consequences of TBI in children. The search was carried out using PubMed and included terms such as “endocrine and traumatic brain injury,” “traumatic brain injury and growth hormone,” “traumatic brain injury and thyroid,” “traumatic brain injury and gonadal axis,” “traumatic brain injury and puberty,” “traumatic brain injury and adrenal axis,” “traumatic brain injury and cortisol,” “traumatic brain injury and ACTH,” “traumatic brain injury and diabetes insipidus,” and “traumatic brain injury and pituitary.”

To be included in the review, studies had to meet two criteria: (1) the study population consisted of children with a confirmed diagnosis of TBI, and (2) participants had undergone some form of endocrine assessment. Studies were excluded if they were not published in English, focused exclusively on adult patients, or did not include endocrine evaluations. The initial screening was performed independently by two reviewers (IC and CC), who assessed the abstracts of all identified articles and applied the inclusion and exclusion criteria. Articles deemed potentially eligible were then examined in full text. Any discrepancies between the reviewers regarding eligibility were resolved through discussion and consensus, ensuring a consistent approach to study selection. Following this process, all studies that met the eligibility criteria were included in the present narrative review and analyzed to provide a comprehensive synthesis of the current evidence regarding endocrine outcomes after pediatric TBI.

## 3. Somatotrophic—IGF1 Axis

Growth hormone (GH), along with its effector insulin-like growth factor 1 (IGF-1), plays a central role in pediatric growth by promoting the differentiation of growth plate precursor cells, which in turn stimulates local IGF-1 production and induces chondrocyte enlargement through both autocrine and paracrine mechanisms [23]. Among the various pituitary hormones, GH is particularly susceptible to disruption following TBI, and children are especially vulnerable due to their ongoing linear growth. The course of GHD after TBI is variable, with some children experiencing transient dysfunction that may resolve over time, while others face a persistent growth impairment. GHD following TBI in children typically develops after a variable latency period, with hormonal disruptions often occurring months to years after the initial injury. This delayed onset is primarily due to the gradual progression of secondary injury to the HPA, including ischemia, inflammation, and potential autoimmune responses. Studies indicate that the majority of children diagnosed with TBI-related GHD show onset of symptoms between 6 months and 2 years post-injury, with some cases taking longer to manifest [24,25,26,27]. Data from the KIMS database revealed that children with TBI were diagnosed with GHD at a younger age compared to those with non-functioning pituitary adenomas (NFPA) [28]. Casano-Sancho’s study found that 11 out of 23 children aged 6 years or older exhibited subnormal GH peaks three months following TBI, with 34% of these cases persisting at one year [29]. In another study, Dassa et al. reported GHD in 17 of 61 children one year after TBI, with one case diagnosed 6.5 years post-injury [27]. The resolution of GHD in pediatric TBI cases is similarly variable, with some children experiencing spontaneous recovery, particularly in the case of mild or transient damage. Longitudinal follow-up studies have indicated that GH secretion may normalize within 1 to 2 years after the injury in some children, especially when the initial injury was less severe and no major structural damage to the HPA occurred [24,27]. Salomon-Estebanez et al. observed that in some cases, IGF-1 levels and growth velocity spontaneously normalized within one year following TBI. Notably, no stimulation testing was necessary, and children showed normal growth throughout the follow-up period, suggesting that milder cases of GHD may resolve without intervention [30]. A longitudinal study by Heather et al., involving follow-up periods ranging from 6.5 to 12 years post-TBI, found that a subset of children with initially subnormal GH levels (8%) showed recovery over time. By the end of the study, these children had regained normal growth velocity and normal IGF-1 levels, indicating that GHD could resolve naturally in some pediatric TBI cases [31]. Similarly, Einaudi et al. reported that among children with moderate TBI, some initially diagnosed with GHD showed improvement in GH secretion within the first two years post-injury. These children either required reduced GH therapy or had fully normalized GH levels, supporting the idea that transient pituitary dysfunction can resolve over time in some children with TBI [18]. In contrast, children with more severe brain injuries or persistent pituitary damage may continue to experience long-term GHD, often requiring lifelong GH replacement therapy. Research suggests that the persistence of GHD beyond two years is more common in cases of significant hypothalamic or pituitary trauma [24,27]. However, there is currently non-conclusive evidence linking Glasgow Coma Scale (GCS) scores or radiological findings with the risk of developing pituitary dysfunction following TBI [2,5,16].

The variability in results across studies regarding GHD following TBI can be primarily attributed to several factors, including the use of different protocols, varying timelines, and inconsistent testing methods. The lack of standardized diagnostic criteria and follow-up protocols has significantly contributed to the heterogeneity of findings [6,29]. For instance, the timing of endocrine assessments varies considerably across studies. Some assess hormonal function shortly after the injury, while others conduct evaluations years later, leading to differences in the identification and diagnosis of GHD. Moreover, the inconsistency in the methods used to diagnose GHD, such as GH stimulation tests and IGF-1 measurements, further contributes to this variability. Different studies have employed various methods to evaluate GH secretion, including the insulin tolerance test (ITT), glucagon stimulation, GHRH–arginine, and clonidine–arginine tests. Other studies, however, have assessed spontaneous overnight GH secretion. Additionally, discrepancies in the GH cut-off values used to diagnose GHD contribute to the lack of uniformity. Some studies use a threshold of 5 ng/mL, typically applied to adults, while others have utilized the 10 ng/mL cut-off previously applied to pediatric patients or the more recent 7 ng/dL threshold set in current pediatric protocols [29]. For example, Norwood et al. reported that, using arginine/glucagon testing, 5 of 32 subjects (16%) failed to achieve an overnight GH peak above 5 ng/mL, while 10 of 32 (22%) had a stimulated peak GH response below 7 ng/mL. In contrast, Heather et al. observed a subnormal GH response of less than 10 mcg/L, with 16 subjects exhibiting a GH peak of less than 5 mcg/L following dual stimulation with clonidine and arginine [2,31]. Another factor contributing to inconsistent findings is the lack of adequate long-term follow-up in many studies, which is essential for detecting delayed or evolving hormonal deficiencies [6,29,32]. Most perspective studies conclude follow-up within one year post-TBI, showing that the majority of children maintained a normal growth despite a high prevalence of abnormal GH stimulation tests. However, some studies, such as those by Poomthavorn et al., included follow-up periods ranging from 1.7 to 9.6 years and found that 72% of children demonstrated normal growth velocity or achieved an appropriate final height within their expected target height range [20,29]. These findings underscore the importance of extended clinical monitoring to fully assess the impact of TBI on growth. Overall, these studies highlight that GHD is a significant and often long-lasting complication following pediatric TBI, requiring prolonged clinical observation. Whilst GHD may be transient assessment should extend beyond 12 months, as early evaluations may fail to capture the full extent of growth impairment. This observation is further supported by recent research in adults, where GH administration has been shown to improve neurological outcomes, likely due to its neurotrophic properties. For instance, Devesa et al. studied the effects of GH treatment in combination with rehabilitation in 13 TBI patients, 5 of whom had acquired GHD. Their findings revealed substantial cognitive improvements, with cognitive function recovery occurring more rapidly and significantly compared to motor function recovery [33]. Similarly, Reimunde et al. investigated the effects of GH therapy combined with cognitive rehabilitation in 11 adults with GHD and cognitive impairments following TBI. The GHD group showed significantly greater improvements in similarities, vocabulary, verbal Intelligence Quotient (IQ), and overall IQ compared to the control group [34].

## 4. Gonadal Axis and Puberty

The regulation of puberty is a highly sensitive and complex biological process that can be significantly affected by TBI. Pubertal development depends on a coordinated sequence of neuroendocrine signals governing both the initiation and progression of puberty; disruptions to this system may result in considerable developmental and also psychological consequences [35,36,37]. Both hypogonadism and central precocious puberty (CPP) have been reported in pediatric populations following TBI.

Children who sustain TBI are more frequently affected by delayed or arrested puberty, commonly due to hypogonadotropic hypogonadism [6,16]. Following GHD, hypogonadism represents one of the most commonly reported endocrine disorders post-TBI. However, its prevalence appears to be lower in pediatric populations, where hypogonadism—typically transient—has been reported in approximately 4–9% of cases, compared to prevalence rates of up to 29% in adult cohorts [16,38,39]. Poomthavorn et al. reported a 4% incidence of hypogonadism within the first year following TBI, with presentations classified as either transient or permanent [20]. Einaudi et al. analyzed two separate cohorts using both retrospective and perspective designs. In the retrospective cohort, hypogonadism was identified in 9% of children between one and four years post-injury, whereas the perspective cohort revealed no cases of hypogonadism at the one-year follow-up [18]. Similarly, Kaulfers et al. found that hypogonadism following TBI in pediatric patients is generally transient. In their study, one male patient developed clinical signs of hypogonadism within the first month post-injury but showed complete recovery by three months. Female patients who experienced secondary amenorrhea also demonstrated resolution within one year. Notably, a new case of hypogonadism was diagnosed at the one-year mark, highlighting the need for ongoing endocrine surveillance for at least 12 months after TBI [40]. This type of hypogonadism results from injury to the hypothalamic GnRH-secreting neurons or the vascular network supplying these regions. Such damage leads to impaired secretion of luteinizing hormone (LH) and follicle-stimulating hormone (FSH), ultimately causing delayed onset or interruption in the progression of secondary sexual characteristics [41,42,43]. It is important to note that elevated prolactin levels can also disrupt the normal pulsatile secretion of GnRH, leading to reduced secretion of LH and FSH, and consequently resulting in impaired gonadal function [7]. In adults, hypogonadism following TBI is often persistent, in contrast to the typically transient dysfunction observed in children. Reported prevalence varies across studies, and most investigations have not found a significant association between gonadotropin deficiency and TBI severity, GCS scores, or CT findings [38,39].

Conversely, CPP has been described in at least ten published cases. For instance, Dassa et al. observed that 4 out of 61 patients (6.6%) developed CPP at a median interval of 5.7 years (range: 2.4–6.1 years) after the traumatic event [6,27]. Similarly, Sockalosky et al. reported CPP onset occurring at mean intervals ranging from 0.4 to 1.6 years post-injury [44]. Importantly, the presence of pituitary dysfunction one year after TBI was significantly associated with increased likelihood of persistent pituitary abnormalities or subsequent CPP more than five years after the initial trauma [6,27]. However, other studies suggest a relatively low incidence of CPP in this population. Heather et al. reported approximately 1–2 cases per pediatric cohort. Among girls, the incidence was comparable to that of the general population (approximately 20 per 10,000), whereas in boys, a slight increase in incidence was observed (approximately 4 per 10,000) [16,31]. The underlying pathophysiological mechanisms of CPP following TBI remain unclear. TBI can disrupt the physiological mechanisms that normally suppress gonadotropin secretion during the prepubertal period. In healthy children, gonadotropin-releasing hormone (GnRH) activity is tightly regulated and actively inhibited through central neural mechanisms [45]. Following TBI, this inhibitory control may be compromised, potentially leading to premature activation of the hypothalamic–pituitary–gonadal (HPG) axis and the onset of CPP [5]. One proposed mechanism involves damage to GABAergic pathways that normally inhibit the GnRH pulse generator. Alternatively, injury to the hypothalamus may impair inhibitory signaling mediated by N-methyl-D-aspartate (NMDA) receptors or glial modulators, further contributing to dysregulation of GnRH secretion. Increased intracranial pressure affecting the hypothalamic–pituitary region may also play a role in disrupting normal inhibitory control over gonadotropin release [35,46,47]. These pathophysiological processes are similar to those observed in other neurological conditions associated with CPP, such as meningitis, hydrocephalus, encephalopathy, or intracranial tumors [5].

## 5. Hypothalamic–Pituitary–Adrenal Axis

A prompt recognition of hypothalamic–pituitary–adrenal axis impairment following TBI is crucial, given its potential to result in life-threatening complications. In acute settings, adrenal insufficiency may be heralded by severe manifestations such as refractory hypotension, hypoglycemia, and hyponatremia. In contrast, chronic forms may emerge insidiously, often months after the injury, with vague symptoms including fatigue, anorexia, diminished stress tolerance, and recurrent abdominal discomfort. These nonspecific features are frequently misattributed to functional or psychological causes, often delaying an appropriate endocrine evaluation until adrenal crisis reveals the underlying deficiency [5,16].

Following TBI, the adrenal axis exhibits a predictable temporal pattern. During the acute phase, serum cortisol levels typically rise in response to stress and neuroinflammation, later declining towards baseline as the injury stabilizes [7,48,49]. Several studies have reported an association between early cortisol levels and injury severity, particularly in mild to moderate TBI, where normalization of cortisol levels has been linked with more favorable outcomes [48,49]. In individuals with preserved hypothalamic–pituitary–adrenal function, early cortisol concentrations generally reflect the degree of physiological stress, with higher levels in more severe injuries [50]. Conversely, a low cortisol level in the context of significant trauma may indicate secondary adrenal insufficiency due to adrenocorticotropic hormone (ACTH) deficiency. In a prospective study, Srinivas et al. evaluated 37 children with severe TBI at 1, 3, and 7 days post-injury. Initial ACTH and cortisol levels were elevated on day 1 but returned to normal by days 3 and 7. ACTH elevation was significantly more frequent in children with fronto-temporal lesions (63% vs. 13%), while a similar but nonsignificant trend was seen for cortisol (63% versus 40%). Interestingly, elevated cortisol was less common in children with the most severe injuries (GCS 3-4). Additionally, 46% of patients showed low cortisol levels and 14% had reduced ACTH values during the follow-up period [51]. Ulutabanca et al. reported pituitary hormone disturbances in 44% out of 41 pediatric patients during the acute phase following TBI, with deficiencies in ACTH in 24% of cases [52].

As the injury evolves, children may shift from a compensated adrenal response to partial or complete hypothalamic–pituitary–adrenal axis failure [7]. This dysfunction can arise at multiple levels: hypothalamic damage may impair corticotropin-releasing hormone (CRH) output, while pituitary injury compromises ACTH production. Additionally, inflammatory mediators such as interleukin-6 (IL-6) may initially stimulate cortisol release, but prolonged exposure can lead to suppression of both CRH and ACTH synthesis. Furthermore, cellular mechanisms like apoptosis and autophagy may contribute to progressive hypothalamic–pituitary damage [7,53,54].

Assessment of hypothalamic–pituitary–adrenal axis integrity post-TBI varies widely among studies, complicating the interpretation of prevalence data. Some authors relied on baseline cortisol levels for screening, limiting dynamic testing to symptomatic or biochemically suggestive cases. Testing protocols also differed, with some studies utilizing low-dose ACTH or glucagon stimulation in place of ITT, which is often contraindicated in pediatric populations. Kaulfers et al. reported no cases of low morning cortisol within six months post-injury, using stringent diagnostic criteria (cortisol <5 µg/dL). Moreover, basal cortisol levels did not consistently predict peak responses to ACTH stimulation. For instance, one patient with a low basal cortisol (3.3 µg/dL) had a normal stimulated value (26.3 µg/dL), while another with normal baseline (11.9 µg/dL) showed a borderline response (18.1 µg/dL) [44]. Similarly, Khadr et al. observed inadequate peak cortisol responses in nine children, including three with significantly reduced stimulated levels, two of whom also had low baseline cortisol [22]. Poomthavorn et al. reported comparable findings: only one out of 15 tested children had a low basal cortisol level, and this patient responded normally to ACTH stimulation [20]. Other studies highlight the transient nature of post-traumatic adrenal dysfunction. Casano-Sancho et al. found that 47% of children had low stimulated cortisol levels at three months post-TBI using the glucagon test, but all but three normalized by 12 months [16]. Heather et al. reported suboptimal cortisol responses in 9% of children, with most normalizing on repeat testing [31]. Bellone et al. evaluated 70 children between 1 and 9 years after TBI and identified four cases of ACTH deficiency, some in combination with other pituitary deficits. At one year, 10% of the cohort had at least one hypothalamic–pituitary hormone abnormality [42]. Additional studies using insulin or glucagon stimulation tests confirmed normalization of the adrenal axis in the majority of children over time [43,48,49].

In summary, while ACTH deficiency may occur following pediatric TBI, it appears less common than GH or gonadotropin deficiencies and is frequently transient. Nevertheless, due to its potentially life-threatening consequences, clinicians should maintain a high index of suspicion and consider temporary glucocorticoid replacement during periods of physiological stress in the first year post-injury.

## 6. Thyroid Axis

Thyroid function abnormalities are frequently seen in the acute phase following brain injury, typically manifesting as a non-thyroidal illness, characterized by reduced levels of triiodothyronine (T3) and low-normal thyroxine (T4), often accompanied by a low-normal or mildly elevated thyroid stimulating hormone (TSH) level. In critically ill patients with acute hypothyroidism, clinical manifestations may include drowsiness, difficulty rousing, bradycardia, heart failure, or hypothermia [5]. The reported prevalence of central hypothyroidism varies depending on how thyroid function is assessed. Kaulfers et al. observed a high prevalence of central hypothyroidism in their cohort, with 46% of patients showing a low TSH response and fT4 levels in the lower part of the normal range [44]. In contrast, other studies have reported a lower prevalence of TSH deficiency, ranging from 5 to 12% [20,30]. The proposed mechanism for disproportionately high TSH in central hypothyroidism involves abnormal glycosylation of the TSH alpha and beta subunits, rendering the secreted TSH biologically inactive [55,56]. Hypothyroidism following TBI can manifest as either a transient or persistent condition. Einaudi et al. reported thyroid dysfunction in seven patients (23%) during the acute post-TBI phase, which completely resolved within six months of follow-up [18]. Similar transient alterations in thyroid function were also described by Casano-Sancho and Kaulfers [16,44]. In contrast, Rao et al. documented a case of persistently reduced TSH concentrations 18 months after injury, suggesting that hypothyroidism—though generally regarded as an acute complication of TBI—may occasionally persist into the chronic phase [57]. Furthermore, Bellone et al. identified a case of TSH deficiency associated with panhypopituitarism among 70 pediatric patients evaluated 1 to 9.1 years after TBI, while Personnier et al. observed central hypothyroidism in six of 87 children assessed at a mean interval of 9.5 ± 3.4 months post-injury [58,59].

Unrecognized and untreated central hypothyroidism may result in impaired growth, chronic fatigue, and increased risk of cardiovascular complications [60,61]. In the absence of specific testing for TSH deficiency, these patients often remain clinically challenging and are left without an appropriate treatment. Reliance solely on free T4 and TSH measurements can fail to identify individuals with impaired TSH secretion. Although overnight dynamic testing represents the most accurate diagnostic approach, it is not feasible as a routine screening tool in children with TBI. A more practical strategy involves measuring TSH levels at 8 a.m. and 4 p.m. in symptomatic patients with free T4 concentrations in the lower third of the reference range. An a.m./p.m. TSH ratio below 1.3 is considered indicative of central hypothyroidism [62].

## 7. Posterior Pituitary Disorders and Fluid Homeostasis

Posterior pituitary dysfunction typically manifests in the acute phase following TBI. Disturbances in water homeostasis, most frequently due to cranial diabetes insipidus (DI), are commonly observed and appear to correlate with injury severity, with patients presenting lower GCS scores at higher risk [63,64]. In the majority of cases, these alterations in water balance are transient and resolve spontaneously within days to weeks after the injury [6]. While DI often develops within hours to days post-injury, its onset may be delayed, sometimes occurring up to 30 days following TBI [6]. Data on post-TBI DI in the pediatric population remain limited. Permanent DI is uncommon, although transient forms have been reported in approximately 4% of affected children [16]. Only a small number of pediatric cases have been documented, and the occurrence of acute DI in children has been associated with a poorer prognosis [6]. For instance, in a series of 19 pediatric patients (aged 4 months to 15 years) who developed acute DI secondary to severe brain injury, only three survived the acute phase [65]. Mariani et al. reported three cases of post-traumatic hypopituitarism in children, two of whom developed DI within days of the injury. In one patient, DI was an isolated defect, whereas in the other it was accompanied by multiple pituitary hormone deficiencies, including growth hormone, thyroid, and gonadal insufficiencies [66].

Overall, although DI can pose serious complications in the acute phase of pediatric TBI, persistent DI appears to be a relatively rare occurrence. Awareness of both transient and, more rarely, long-lasting posterior pituitary dysfunction is important for timely diagnosis, monitoring, and management in affected children.

## 8. Future Directions and the Need for Endocrine Assessment

The recognition of HPA dysfunction following pediatric TBI has underscored the critical need for structured and longitudinal endocrine monitoring in this vulnerable population. Despite considerable advances in understanding the pathophysiology of post-traumatic endocrine disorders, significant gaps remain regarding their true incidence, natural history, and long-term clinical impact. Reported prevalence rates vary widely across studies due to differences in injury severity, timing of assessments, diagnostic methodologies, and duration of follow-up, highlighting the urgent need for standardized protocols for endocrine evaluation. In pediatric TBI, there is an urgent need to develop a predictive model for post-injury endocrine dysfunction. Early identification of at-risk children—based on injury characteristics, clinical features, imaging, and biochemical markers—could enable timely interventions, mitigate long-term developmental and metabolic consequences, and reduce the prevalence of undiagnosed hormonal deficits. A conceptual predictive model for post-TBI endocrine dysfunction in pediatric patients could integrate multiple domains to stratify risk. First, the severity of the injury (mild, moderate, or severe) would serve as a primary determinant. Patient’s age could modulate susceptibility, given developmental differences in the hypothalamic–pituitary axis. The anatomical localization of brain injury, particularly involvement of the hypothalamus or pituitary, would provide additional specificity. Clinical and laboratory biomarkers, including baseline hormone levels and inflammatory markers, could offer early biochemical indicators of dysfunction. Finally, additional clinical factors—such as time elapsed since trauma, pre-existing comorbidities, and concomitant medications—would refine risk prediction. By combining these variables into a weighted scoring system or machine learning-based framework, the model could generate individualized risk profiles, guiding targeted endocrine evaluation and follow-up.

**Figure 2:**
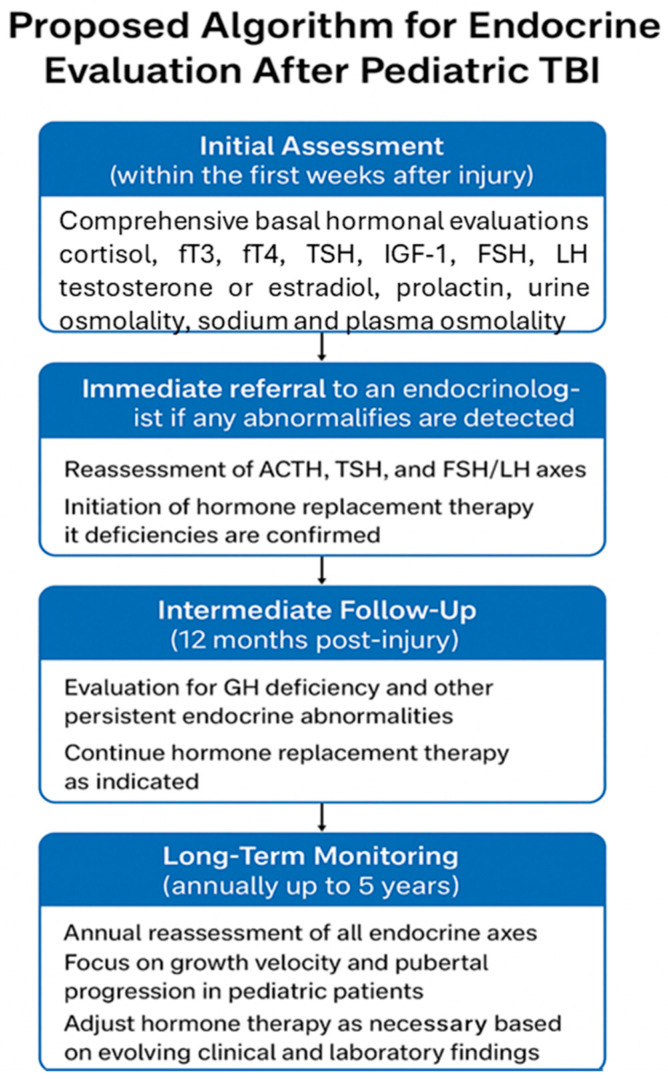
Hypothetical algorithm for hormonal assessment after traumatic brain injury.

Future research should focus on developing evidence-based screening strategies that define the optimal timing and frequency of hormonal assessments, identify high-risk populations, and establish early biomarkers predictive of long-term dysfunction. Particular attention should be directed toward axes that are highly susceptible to injury, including GH, gonadal, thyroid, adrenal, and posterior pituitary systems. Understanding the temporal evolution of these hormonal disturbances is essential, as many deficiencies—including GHD, hypogonadism, central hypothyroidism, and subtle posterior pituitary dysfunction—may manifest months to years after the initial insult, often with insidious clinical presentations. Without systematic monitoring, these conditions may remain unrecognized, leading to preventable morbidities such as impaired growth, delayed or arrested puberty, metabolic dysregulation, fatigue, and reduced quality of life. From a clinical perspective, the integration of comprehensive endocrine assessment into both the acute and long-term follow-up of pediatric moderate and severe TBI patients is imperative. This includes baseline and serial measurements of relevant hormones, dynamic stimulation testing when indicated, and careful monitoring of growth and pubertal milestones. Early identification of endocrine deficits allows timely intervention, including hormone replacement therapy, which has the potential not only to restore normal physiological function but also to improve neurocognitive recovery and overall rehabilitation outcomes. Routine basal hormonal screening for TBI-induced hypopituitarism should include serum cortisol, free T3, free T4, TSH, IGF-1, FSH, LH, testosterone (in boys) or estradiol (in girls), prolactin, urine specific gravity, sodium, and plasma osmolality. Patients should be referred to an endocrinologist for dynamic stimulation testing if the results of their initial evaluations are inconclusive or indicate the need for further assessment [32]. Although a precise timing for endocrine evaluation in the pediatric population has not been established by guidelines, available evidence suggests that a comprehensive endocrine assessment should be performed within the first weeks after the acute event, at 6 months, and at 12 months, followed by annual follow-up for at least five years [5]. A potential algorithm, as proposed by this review, is reported in Figure 2.

Collaborative multicenter studies and establishing pediatric TBI endocrine registries may represent promising avenues for future research. Such initiatives could facilitate large-scale longitudinal data collection, enabling the identification of risk factors for specific pituitary deficits, the natural course of transient versus permanent dysfunction, and the long-term impact of therapeutic interventions. Additionally, mechanistic studies examining the contributions of ischemia, inflammation, structural injury, and potential autoimmune processes to post-traumatic hypopituitarism will be essential to refine preventive and therapeutic strategies. In summary, a proactive approach to endocrine evaluation in pediatric TBI is essential to mitigate the long-term consequences of HPA dysfunction. Implementing standardized, longitudinal assessment protocols, coupled with targeted therapeutic interventions, has the potential to substantially improve growth, pubertal development, metabolic health, and quality of life of affected children. Future research must continue to elucidate the pathophysiology, natural history, and optimal management strategies for post-traumatic endocrine disorders, ensuring that these silent yet impactful complications are neither overlooked nor undertreated.

## 9. Conclusions

Pediatric TBI is a leading cause of morbidity and long-term disability in children, with endocrine disturbances emerging as a significant yet often underappreciated consequence. The HPA is particularly susceptible to injury, and post-traumatic endocrine dysfunction may involve multiple hormonal axes, including GH, gonadotropins, thyroid, adrenal, and posterior pituitary hormones. Each axis exhibits differential vulnerability, distinct temporal patterns of onset, and variable courses of recovery or persistence, reflecting the complex pathophysiology of TBI in the pediatric population. GHD is the most frequently reported anterior pituitary disorder following pediatric TBI. Clinical manifestations range from transient impairment to persistent deficiency, which may appear months to years after the initial insult. This variability underscores the need for long-term endocrine monitoring, individualized according to factors such as injury severity, mechanism, and age at the time of trauma. Gonadal axis abnormalities, including hypogonadism and, less commonly, central precocious puberty, further illustrate the impact of TBI on pubertal development and future reproductive function, with potential consequences for psychosocial well-being. Early identification and timely intervention are therefore crucial. While adrenal and thyroid dysfunctions are less frequently reported, they may have life-threatening implications if unrecognized. Adrenal insufficiency can present acutely or insidiously, often with subtle symptoms, whereas hypothyroidism can adversely affect growth, cognitive function, and metabolic homeostasis. Disorders of the posterior pituitary, particularly DI, usually arise in the acute post-injury period and are frequently transient; however, persistent forms may occur, particularly following severe TBI or direct hypothalamic involvement. The prevalence, timing, and severity of post-TBI endocrine dysfunction in children are highly variable, influenced by differences in injury characteristics, diagnostic approaches, and follow-up duration. The often delayed and insidious onset of many hormonal deficits highlights the importance of structured, long-term surveillance to identify both transient and permanent impairments. Prompt recognition and individualized management of post-traumatic endocrine sequelae are essential not only to optimize growth, pubertal progression, and metabolic outcomes, but also to improve overall quality of life in pediatric TBI survivors. Future research should prioritize: (1) the development and validation of standardized screening protocols for high-risk populations, (2) the investigation of mechanisms underlying pituitary vulnerability, and (3) the assessment of long-term efficacy and safety of hormone replacement therapies, including GH and gonadal steroids, in improving growth, metabolic regulation, and psychosocial outcomes. Moreover, multi-center prospective studies with standardized endocrine evaluation schedules are needed to better define the natural history, risk factors, and optimal management strategies for post-TBI endocrine dysfunction in children.

## Data Availability

Not applicable.

## References

[1] Srinivas R., Brown S.D., Chang Y.F., Garcia-Fillion P., Adelson P.D. (2010). Endocrine function in children acutely following severe traumatic brain injury. Child’s Nerv. Syst..

[2] Norwood K.W., Deboer M.D., Gurka M.J., Kuperminc M.N., Rogol A.D., Blackman J.A., Wamstad J.B., Buck M.L., Patrick P.D. (2010). Traumatic brain injury in children and adolescents: Surveillance for pituitary dysfunction. Clin. Pediatr..

[3] Di Sarno L., Curatola A., Cammisa I., Capossela L., Eftimiadi G., Gatto A., Chiaretti A. (2022). Non-pharmacologic approaches to neurological stimulation in patients with severe brain injuries: A systematic review. Eur. Rev. Med. Pharmacol. Sci..

[4] Ferrara P., Cammisa I., Zona M., Corsello G., Giardino I., Vural M., Bali D., Pastore M., Pettoello-Mantovani M. (2024). Child Opportunity Index: A Multidimensional Indicator to Measure Neighborhood Conditions Influencing Children’s Health. J. Pediatr..

[5] Reifschneider K., Auble B.A., Rose S.R. (2015). Update of Endocrine Dysfunction following Pediatric Traumatic Brain Injury. J. Clin. Med..

[6] Acerini C.L., Tasker R.C., Bellone S., Bona G., Thompson C.J., Savage M.O. (2006). Hypopituitarism in childhood and adolescence following traumatic brain injury: The case for prospective endocrine investigation. Eur. J. Endocrinol..

[7] Richmond E., Rogol A.D. (2014). Traumatic brain injury: Endocrine consequences in children and adults. Endocrine.

[8] Ntali G., Tsagarakis S. (2019). Traumatic brain injury induced neuroendocrine changes: Acute hormonal changes of anterior pituitary function. Pituitary.

[9] Tanriverdi F., De Bellis A., Battaglia M., Bellastella G., Bizzarro A., Sinisi A.A., Bellastella A., Unluhizarci K., Selcuklu A., Casanueva F.F. (2010). Investigation of antihypothalamus and antipituitary antibodies in amateur boxers: Is chronic repetitive head trauma-induced pituitary dysfunction associated with autoimmunity?. Eur. J. Endocrinol..

[10] Tanriverdi F., De Bellis A., Bizzarro A., Sinisi A.A., Bellastella G., Pane E., Bellastella A., Unluhizarci K., Selcuklu A., Casanueva F.F. (2008). Antipituitary antibodies after traumatic brain injury: Is head trauma-induced pituitary dysfunction associated with autoimmunity?. Eur. J. Endocrinol..

[11] Wachter D., Gündling K., Oertel M.F., Stracke H., Böker D.K. (2009). Pituitary insufficiency after traumatic brain injury. J. Clin. Neurosci..

[12] Salehi F., Kovacs K., Scheithauer B.W., Pfeifer E.A., Cusimano M. (2007). Histologic study of the human pituitary gland in acute traumatic brain injury. Brain Inj..

[13] Ferrara P., Cammisa I., Zona M., Pacucci I., Grimaldi M.T., Scaltrito F., Giardino I., Verrotti A., Pettoello-Mantovani M. (2025). Child Maltreatment and Psychopathology: A Brief Review on the Potential Role of the Hypothalamic-pituitary-adrenal Axis. Curr. Pediatr. Rev..

[14] Grossman W.F., Sanfield J.A. (1994). Hypothalamic atrophy presenting as amenorrhea and sexual infantilism in a female adolescent. A case report. J. Reprod. Med..

[15] Yamanaka C., Momoi T., Fujisawa I., Kikuchi K., Kaji M., Sasaki H., Yorifuji T., Mikawa H. (1993). Acquired growth hormone deficiency due to pituitary stalk transection after head trauma in childhood. Eur. J. Pediatr..

[16] Casano-Sancho P. (2017). Pituitary dysfunction after traumatic brain injury: Are there definitive data in children?. Arch. Dis. Child..

[17] Lauzier F., Turgeon A.F., Boutin A., Shemilt M., Côté I., Lachance O., Archambault P.M., Lamontagne F., Moore L., Bernard F. (2014). Clinical outcomes, predictors, and prevalence of anterior pituitary disorders following traumatic brain injury: A systematic review. Crit. Care Med..

[18] Einaudi S., Matarazzo P., Peretta P., Grossetti R., Giordano F., Altare F., Bondone C., Andreo M., Ivani G., Genitori L. (2006). Hypothalamo-hypophysial dysfunction after traumatic brain injury in children and adolescents: A preliminary retrospective and prospective study. J. Pediatr. Endocrinol. Metab..

[19] Niederland T., Makovi H., Gál V., Andréka B., Abrahám C.S., Kovács J. (2007). Abnormalities of pituitary function after traumatic brain injury in children. J. Neurotrauma.

[20] Poomthavorn P., Maixner W., Zacharin M. (2008). Pituitary function in paediatric survivors of severe traumatic brain injury. Arch. Dis. Child..

[21] Moon R.J., Sutton T., Wilson P.M., Kirkham F.J., Davies J.H. (2010). Pituitary function at long-term follow-up of childhood traumatic brain injury. J. Neurotrauma.

[22] Khadr S.N., Crofton P.M., Jones P.A., Wardhaugh B., Roach J., Drake A.J., Minns R.A., Kelnar C.J. (2010). Evaluation of pituitary function after traumatic brain injury in childhood. Clin. Endocrinol..

[23] Cammisa I., Rigante D., Cipolla C. (2025). A Theoretical Link Between the GH/IGF-1 Axis and Cytokine Family in Children: Current Knowledge and Future Perspectives. Children.

[24] Wexler T.L., Reifschneider K., Backeljauw P., Cárdenas J.F., Hoffman A.R., Miller B.S., Yuen K.C.J. (2023). Growth Hormone Deficiency Following Traumatic Brain Injury in Pediatric and Adolescent Patients: Presentation, Treatment, and Challenges of Transitioning from Pediatric to Adult Services. J. Neurotrauma.

[25] Yuen K.C.J., Masel B.E., Reifschneider K.L., Sheffield-Moore M., Urban R.J., Pyles R.B. (2020). Alterations of the GH/IGF-I Axis and Gut Microbiome after Traumatic Brain Injury: A New Clinical Syndrome?. J. Clin. Endocrinol. Metab..

[26] Soliman A.T., Adel A., Soliman N.A., Elalaily R., De Sanctis V. (2015). Pituitary Deficiency Following Traumatic Brain Injury in Early Childhood: A Review of the Literature. Georgian Med. News.

[27] Dassa Y., Crosnier H., Chevignard M., Viaud M., Personnier C., Flechtner I., Meyer P., Puget S., Boddaert N., Breton S. (2019). Pituitary deficiency and precocious puberty after childhood severe traumatic brain injury: A long-term follow-up prospective study. Eur. J. Endocrinol..

[28] Gardner C.J., Mattsson A.F., Daousi C., Korbonits M., Koltowska-Haggstrom M., Cuthbertson D.J. (2015). GH deficiency after traumatic brain injury: Improvement in quality of life with GH therapy: Analysis of the KIMS database. Eur. J. Endocrinol..

[29] Casano-Sancho P., Suárez L., Ibáñez L., García-Fructuoso G., Medina J., Febrer A. (2013). Pituitary dysfunction after traumatic brain injury in children: Is there a need for ongoing endocrine assessment?. Clin. Endocrinol..

[30] Salomón-Estébanez M.A., Grau G., Vela A., Rodríguez A., Morteruel E., Castaño L., Rica I. (2014). Is routine endocrine evaluation necessary after paediatric traumatic brain injury?. J. Endocrinol. Investig..

[31] Heather N.L., Jefferies C., Hofman P.L., Derraik J.G., Brennan C., Kelly P., Hamill J.K., Jones R.G., Rowe D.L., Cutfield W.S. (2012). Permanent hypopituitarism is rare after structural traumatic brain injury in early childhood. J. Clin. Endocrinol. Metab..

[32] Ghigo E., Masel B., Aimaretti G., Léon-Carrión J., Casanueva F.F., Dominguez-Morales M.R., Elovic E., Perrone K., Stalla G., Thompson C. (2005). Consensus guidelines on screening for hypopituitarism following traumatic brain injury. Brain Inj..

[33] Devesa J., Reimunde P., Devesa P., Barberá M., Arce V. (2013). Growth hormone (GH) and brain trauma. Horm. Behav..

[34] Reimunde P., Quintana A., Castañón B., Casteleiro N., Vilarnovo Z., Otero A., Devesa A., Otero-Cepeda X.L., Devesa J. (2011). Effects of growth hormone (GH) replacement and cognitive rehabilitation in patients with cognitive disorders after traumatic brain injury. Brain Inj..

[35] De Sanctis V., Soliman A.T., Elsedfy H., Soliman N.A., Elalaily R., El Kholy M. (2015). Precocious Puberty Following Traumatic Brain Injury in Early Childhood: A Review of the Literature. Pediatr. Endocrinol. Rev..

[36] Livny A., Silberg T. (2023). Puberty, brain network connectivity and neuropsychiatric outcomes following pediatric traumatic brain injury in females: A research protocol. PLoS ONE.

[37] De Sanctis V., Sprocati M., Govoni M.R., Raiola G. (2008). Assessment of traumatic brain injury and anterior pituitary dysfunction in adolescents. Georgian Med. News.

[38] Hohl A., Mazzuco T.L., Coral M.H., Schwarzbold M., Walz R. (2009). Hypogonadism after traumatic brain injury. Arq. Bras. Endocrinol. Metabol..

[39] Agha A., Thompson C.J. (2005). High risk of hypogonadism after traumatic brain injury: Clinical implications. Pituitary.

[40] Kaulfers A.M., Backeljauw P.F., Reifschneider K., Blum S., Michaud L., Weiss M., Rose S.R. (2010). Endocrine dysfunction following traumatic brain injury in children. J. Pediatr..

[41] Sodero G., Cipolla C., Rigante D., Arzilli F., Mercuri E.M. (2025). Pubertal induction therapy in pediatric patients with Duchenne muscular dystrophy. J. Pediatr. Endocrinol. Metab..

[42] Sav A., Rotondo F., Syro L.V., Serna C.A., Kovacs K. (2019). Pituitary pathology in traumatic brain injury: A review. Pituitary.

[43] Villa P., Cipolla C., Amar I., Sodero G., Pane L.C., Ingravalle F., Pontecorvi A., Scambia G. (2024). Bone mineral density and body mass composition measurements in premenopausal anorexic patients: The impact of lean body mass. J. Bone Miner Metab..

[44] Sockalosky J.J., Kriel R.L., Krach L.E., Sheehan M. (1987). Precocious puberty after traumatic brain injury. J. Pediatr..

[45] Cammisa I., Malavolta E., Arzilli F., Rotunno G., Rigante D., Cipolla C. (2025). The Effect of GnRH Analogs on Body Mass Index in Girls with Central Precocious Puberty: A Single-Center Retrospective Study with a Literature Review. Children.

[46] Grumbach M.M. (2002). The neuroendocrinology of human puberty revisited. Horm. Res..

[47] Bourguignon J.P., Gérard A., Purnelle G., Czajkowski V., Yamanaka C., Lemaître M., Rigo J.M., Moonen G., Franchimont P. (1997). Duality of glutamatergic and GABAergic control of pulsatile GnRH secretion by rat hypothalamic explants: II. Reduced NR2C- and GABAA-receptor-mediated inhibition at initiation of sexual maturation. J. Neuroendocrinol..

[48] Cernak I., Savic V.J., Lazarov A., Joksimovic M., Markovic S. (1999). Neuroendocrine responses following graded traumatic brain injury in male adults. Brain Inj..

[49] Barton R.N., Stoner H.B., Watson S.M. (1987). Relationships among plasma cortisol, adrenocorticotrophin, and severity of injury in recently injured patients. J. Trauma.

[50] Woolf P.D., Cox C., Kelly M., Nichols D., McDonald J.V., Hamill R.W. (1990). The adrenocortical response to brain injury: Correlation with the severity of neurologic dysfunction, effects of intoxication, and patient outcome. Alcohol. Clin. Exp. Res..

[51] Sodero G., Cipolla C., Camporesi A., Martino L., Costa S., Cannioto Z., Frassanito P., Tamburrini G., Veredice C., Maggio L. (2025). Endocrinologic Dysfunctions and Neuropsychiatric Sequelae in Pediatric Patients with a History of Central Nervous System Infection (ENDLESS): A Prospective Monocentric Study. Pediatr. Infect. Dis. J..

[52] Ulutabanca H., Hatipoglu N., Tanriverdi F., Gökoglu A., Keskin M., Selcuklu A., Kurtoglu S., Kelestimur F. (2014). Prospective investigation of anterior pituitary function in the acute phase and 12 months after pediatric traumatic brain injury. Child’s Nerv. Syst..

[53] Taheri S., Karaca Z., Mehmetbeyoglu E., Hamurcu Z., Yilmaz Z., Dal F., Çınar V., Ulutabanca H., Tanriverdi F., Unluhizarci K. (2022). The Role of Apoptosis and Autophagy in the Hypothalamic-Pituitary-Adrenal (HPA) Axis after Traumatic Brain Injury (TBI). Int. J. Mol. Sci..

[54] Zhang M., Han X., Yan L., Fu Y., Kou H., Shang C., Wang J., Liu H., Jiang C., Wang J. (2024). Inflammatory response in traumatic brain and spinal cord injury: The role of XCL1-XCR1 axis and T cells. CNS Neurosci. Ther..

[55] Faglia G., Bitensky L., Pinchera A., Ferrari C., Paracchi A., Beck-Peccoz P., Ambrosi B., Spada A. (1979). Thyrotropin secretion in patients with central hypothyroidism: Evidence for reduced biological activity of immunoreactive thyrotropin. J. Clin. Endocrinol. Metab..

[56] Hanley P., Lord K., Bauer A.J. (2016). Thyroid Disorders in Children and Adolescents: A Review. JAMA Pediatr..

[57] Rao A., Laghari A.A., Bari I., Khalid M.U., Kirmani S., Bari M.E. (2023). Endocrine Abnormalities in Children with Traumatic Brain Injury at a Tertiary Care Center. Cureus.

[58] Bellone S., Einaudi S., Caputo M., Prodam F., Busti A., Belcastro S., Parlamento S., Zavattaro M., Verna F., Bondone C. (2013). Measurement of height velocity is an useful marker for monitoring pituitary function in patients who had traumatic brain injury. Pituitary.

[59] Personnier C., Crosnier H., Meyer P., Chevignard M., Flechtner I., Boddaert N., Breton S., Mignot C., Dassa Y., Souberbielle J.C. (2014). Prevalence of pituitary dysfunction after severe traumatic brain injury in children and adolescents: A large prospective study. J. Clin. Endocrinol. Metab..

[60] Cammisa I., Rigante D., Cipolla C. (2024). Growth Outcomes and Final Height in Children with Acquired Hypothyroidism: A Systematic Review. Children.

[61] Singh S., Duggal J., Molnar J., Maldonado F., Barsano C.P., Arora R. (2008). Impact of subclinical thyroid disorders on coronary heart disease, cardiovascular and all-cause mortality: A meta-analysis. Int. J. Cardiol..

[62] Rose S.R. (2010). Improved diagnosis of mild hypothyroidism using time-of-day normal ranges for thyrotropin. J. Pediatr..

[63] Nemergut E.C., Zuo Z., Jane J.A., Laws E.R. (2005). Predictors of diabetes insipidus after transsphenoidal surgery: A review of 881 patients. J. Neurosurg..

[64] Barzilay Z., Somekh E. (1988). Diabetes insipidus in severely brain damaged children. J. Med..

[65] Agha A., Sherlock M., Phillips J., Tormey W., Thompson C.J. (2005). The natural history of post-traumatic neurohypophysial dysfunction. Eur. J. Endocrinol..

[66] Mariani R., Bortoluzzi M.N., Richelme C., El Barbary M., Coussement A. (1996). Hypopituitarisme post-traumatisme crânien: À propos de trois cas. Arch. Pediatr..

